# Making a difference: 5 years of Cardiac Surgery Intersociety Alliance (CSIA)

**DOI:** 10.1093/ejcts/ezae048

**Published:** 2024-06-10

**Authors:** R M Bolman, P Zilla, F Beyersdorf, P Boateng, J Bavaria, J Dearani, J Pomar, S Kumar, T Chotivatanapong, K Sliwa, J L Eisele, Z Enumah, B Podesser, E A Farkas, T Kofidis, L J Zühlke, R Higgins

**Affiliations:** Division of Cardio-Thoracic Surgery, Department of Surgery, University of Minnesota, Minneapolis, Minnesota, USA; Christiaan Barnard Department of Cardiothoracic Surgery, Groote Schuur Hospital, University of Cape Town, Cape Town, South Africa; Department of Cardiovascular Surgery, University Hospital Freiburg, Medical Faculty of the Albert-Ludwigs-University, Freiburg, Germany; Department of Cardiovascular Surgery, Icahn School of Medicine, Mount Sinai (ISMMS) Medical Center, New York, NY, USA; Division of Cardiovascular Surgery, Penn Medicine and Heart and Vascular Center, University of Pennsylvania, Philadelphia, PA, USA; Department of Cardiovascular Surgery, Mayo Clinic, Rochester, MN, USA; Department of Cardiovascular Surgery, University of Barcelona, Barcelona, Spain; Department of Cardiovascular and Thoracic Surgery, All India Institute for Medical Sciences, Delhi, India; Department of Cardiothoracic Surgery, Central Chest Institute of Thailand and, Bangkok Heart Center, Bangkok, Thailand; Cape Heart Institute and Division of Cardiology, University of Cape Town, Cape Town, South Africa; World Heart Federation (WHF), Geneva, Switzerland; Department of General Surgery, Johns Hopkins School of Medicine, Baltimore, MD, USA; Center for Biomedical Research and Translational Medicine, University of Vienna, Vienna, Austria; Department of Cardiothoracic Surgery, University Hospital St. Pölten, St. Pölten, Austria; Division of Cardiothoracic Surgery, Indiana University School of Medicine, Indianapolis, IN, USA; Department of Cardiac-, Thoracic- and Vascular Surgery, National Univ. Hospital of Singapore, Singapore; South African Medical Research Council, Cape Town, South Africa; Brigham and Women’s Hosp. and Mass General Hospital, Harvard University, Boston, MA, USA

**Keywords:** Sustainable cardiac surgery, Developing countries

## Abstract

Informed by the almost unimaginable unmet need for cardiac surgery in the developing regions of the world, leading surgeons, cardiologists, editors in chief of the major cardiothoracic journals as well as representatives of medical industry and government convened in December 2017 to address this unacceptable disparity in access to care. The ensuing “Cape Town Declaration” constituted a clarion call to cardiac surgical societies to jointly advocate the strengthening of sustainable, local cardiac surgical capacity in the developing world. The Cardiac Surgery Intersociety Alliance (CSIA) was thus created, comprising The Society of Thoracic Surgeons (STS), the American Association for Thoracic Surgery (AATS), the Asian Society for Cardiovascular and Thoracic Surgery (ASCVTS), the European Association for Cardio-Thoracic Surgery (EACTS) and the World Heart Federation (WHF). The guiding principle was advocacy for sustainable cardiac surgical capacity in low-income countries. As a first step, a global needs assessment confirmed rheumatic heart disease as the overwhelming pathology requiring cardiac surgery in these regions. Subsequently, CSIA published a request for proposals to support fledgling programmes that could demonstrate the backing by their governments and health care institution. Out of 11 applicants, and following an evaluation of the sites, including site visits to the 3 finalists, Mozambique and Rwanda were selected as the first Pilot Sites. Subsequently, a mentorship and training agreement was completed between Mozambique and the University of Cape Town, a middle-income country with a comparable burden of rheumatic heart disease. The agreement entails regular video calls between the heart teams, targeted training across all aspects of cardiac surgery, as well as on-site presence of mentoring teams for complex cases with the strict observance of ‘assisting only’. In Rwanda, Team Heart, a US and Rwanda-based non-governmental organization (NGO) that has been performing cardiac surgery in Rwanda and helping to train the cardiac surgery workforce since 2008, has agreed to continue providing mentorship for the local team and to assist in the establishment of independent cardiac surgery with all that entails. This involves intermittent virtual conferences between Rwandan and US cardiologists for surgical case selection. Five years after CSIA was founded, its ‘Seal of Approval’ for the sustainability of endorsed programmes in Mozambique and Rwanda has resulted in higher case numbers, a stronger government commitment, significant upgrades of infrastructure, the nurturing of generous consumable donations by industry and the commencement of negotiations with global donors for major grants. Extending the CSIA Seal to additional deserving programmes could further align the international cardiac surgical community with the principle of local cardiac surgery capacity-building in developing countries.

## BACKGROUND

Large parts of the world have still no access to life-saving heart surgery [1–5]. Moreover, in the public perception, the need for cardiac surgery (CS) in developing countries has always paled in the face of the infectious diseases challenges, exacerbated by the setting of severe resource limitations. Furthermore, fly-in missions by non-governmental organizations (NGOs) [6, 7]—primarily for children—often gave the impression in some circles that the most urgent needs were taken care of [8]. At the same time, the dramatically diminishing incidence of rheumatic heart disease (RHD) in high-income countries (HICs) was erroneously interpreted as a worldwide development. In the absence of reliable government and/or public health statistics in affected regions, however, the notion of RHD being a disease of the past and coronary artery disease being a disease of the future further kept the perception alive that, in developing countries, adult CS was not the most pressing matter [9]. In this scenario, with the countries hampered by poorly funded healthcare systems, overwhelmed by infectious diseases including HIV, neither the need-assessment for open-heart surgery nor the allocation of the necessary resources were healthcare priorities.

In light of these circumstances, public awareness for the surgical needs of RHD had faded by the time cardiology societies eventually embraced the issue [10, 11]. Such discipline-specific engagements by professional organizations had always played a crucial role in the response of the greater society to health challenges [12–15]. Initially driven by African cardiologists [10, 11, 16, 17], RHD had slowly moved back onto the global research agenda [11, 18, 19] with key data emerging largely from RHD-endemic countries.

The ‘Global Burden of Disease’ estimated that in 2023, there were over 55 million people living with this disease: 10.7 million DALYS (Disability-adjusted Life Years) and >396 000 deaths annually [20]. The increase in prevalence suggests survivor bias, although this is limited to regions with surgical and medical expertise. New datasets on the genetic predisposition to RHD and proteomic signatures represent potential avenues for future interventions [21].

Eventually, sustained multi-pronged advocacy [17] led to the 2018 resolution by the World Health Organisation on Rheumatic Fever and Rheumatic Heart Disease, declaring it a global health priority [22]. This declaration set the stage for the emergence of momentum for a global response [17, 18, 23]. In the wake of these developments, tangible progress was made in primary and secondary prophylaxis with the landmark GOAL clinical trial [24] and the publication of the revised World Heart Federation (WHF) echo screening guidelines [25] and poor 12-month outcomes [26] in low-income countries (LICs). From disease progression studies in Africa [27] to the ambitious and resource-intense development of a vaccine by the Australian and New Zealand Governments [28] to major non-communicable disease initiatives [29], RHD re-emerged as a top research and health policy concern. Reflecting this new impetus, multi-national clinical studies like the ‘INVICTUS’ study have already created unprecedented data sets for tailor-made treatment regimens in RHD [30] outlining the need for clearer actions regarding tertiary interventions such as surgery [31].

However, while the final goal of this increased focus on RHD [17–19, 22, 32, 33] is preventing RHD in the 1st place, it also became clear that, due to the protracted course of the disease, a large number of patients will continue to require surgery for years to come [19, 32, 34]. Unfortunately, while the prevalence of asymptomatic RHD could be determined in screening programmes [35], the proportion of patients actually needing surgery as a consequence of failed prophylaxis remained unknown. Moreover, without the possibility of surgical referral, there was little impetus for prevalence studies in countries lacking CS [4, 5, 36].

Faced with this growing engagement of cardiology societies for RHD [10, 11, 19], it became increasingly imperative for the global professional associations of CS to also unite behind the cause and take on an activist role. Time seemed ripe to get on board for a multitude of reasons. For 1, several governments have eventually realized the cost effectiveness of heart valve surgery, as it largely restores health at the peak of a person’s productivity [34], often leading to a return to a productive role in society. Addressing this need, significant initiatives in global CS began to emerge, including externally funded infrastructure projects. On the skills side, resident training has begun to shift from the USA and Europe to India and China with their more representative incidences of RHD.

Most promisingly, ground-breaking new technologies have already made tele-medicine a reality. Cellphone-based echo capabilities [37], for instance, and internet-based group-communications, help to better improve communication for the few existing cardiac centres. New drug developments like direct oral anticoagulants may create a new urgency for valve repairs in patients with RHD [38, 39] and atrial fibrillation [40]. Furthermore, transcatheter approaches [41, 42] and affordable modern imaging [43] have the potential to be transformative in the future.

In recognition of the urgency for the CS community to meaningfully engage in these re-emphasized global dynamics, representatives of the major cardiothoracic surgical societies, the editors in chief of their journals and the World Heart Federation undertook to:

- unite behind a call-to-arms to advocate sustainable CS capacity-building in LICs [1, 44–49].
- determine the magnitude of the problem by a jointly conducted surgical need assessment for RHD in low- to middle-income countries (LMICs) [2].
- lend its ‘seal of approval’ to pilot programmes subscribing to the principles of sustainability on the basis of a commitment by the local government and institution; mentorship and training agreements with leading institutions in middle-income countries that reflect the rheumatic predominance of their own environment and long-term academic partnerships with tertiary institutions in industrialized countries [50].

The ensuing umbrella structure is called Cardiac Surgery Intersociety Alliance (CSIA) [50–52]. This is a report 5 years after CSIA has been ratified by all participating societies.

## PRINCIPLES AND IMPLEMENTATION OF CARDIAC SURGERY INTERSOCIETY ALLIANCE

### Origins of Cardiac Surgery Intersociety Alliance

At the 50th anniversary of the 1st heart transplant in 2017, a broad consensus was reached among representatives of all major CS societies, the editors of their leading journals and the World Heart Federation that cardiac surgical initiatives in developing countries should henceforth be focussed on the establishment of sustainable local operating capacity [18, 53].

Culminating in the ‘Cape Town Declaration’ (CTD) [1, 44–49], the adoption of this principle inevitably represented a departure from the prevailing enthusiasm for fly-in missions, where an entire team of overseas specialists operates a small number of patients.

To this end, the CSIA called for in the CTD was initiated and endorsed by each of the participating societies. In spite of the unforeseen and difficult obstacles posed by the pandemic, CSIA successfully concludes its first 5 years as a firmly established entity:

- Councils of all 5 major cardiac surgical societies (USA, Asia and Europe) and of the World Heart Federation ‘ratified’ their CSIA participation in 2018/2019. The founding document dedicates the alliance to fostering local cardiac surgical capacity in the developing countries. The initial mandate is to address the neglected yet predominant cardiac pathology of RHD.
- CSIA’s ‘administrative home’ at the University of Cape Town (UCT) was carefully selected as a location in a middle-income country where high standards of CS coincide with a high burden of RHD.

### Needs assessment

As a first step, signatories of the CTD representing LMICs with a combined population of 1.6 billion people engaged in a needs-assessment for surgery for RHD to grasp the true extent of unmet heart valve interventions in these regions [2, 3]. The study was the first to assess the proportion of patients previously diagnosed in RHD screening studies [35] who ultimately require surgery. It showed that, contrary to the widely perceived downward trend of RHD, it remains the single most common cardiovascular disease in young adult and adolescent patients in need of heart surgery in low- and lower-middle-income countries, outweighing other indications such as congenital cardiac defects almost 4-fold [3]. The overall need ranged from 200–250 operations/million population in endemic regions of South Asia and Sub-Saharan Africa to 300–400 operations/million population in endemic hot-spots like Oceania [2, 3]. The study also revealed that only 0.5–7 cardiac operations/million are actually provided in LICs compared to >1000 operations/million in industrialized countries [2, 3].

This dismally low level of CS stands in stark contrast to the often optimistic assessment of NGOs regarding the impact of their ‘missions’ [54, 55]. What aggravates the latter is the disproportional focus on paediatric cases. Of the 86 NGOs actively providing CS in LMICs in 2022, only 5 (6%) focused on adult CS (i.e. RHD), 25 (29%) performed at least some degree of adult open-heart surgery and 56 (65%) performed exclusively paediatric CS [56], although adult and adolescent patients with their predominant RHD are more than 4 times more common than children with heart defects [3]. Moreover, the sophisticated post-operative follow-up adults with congenital heart disease often require for life is non-existent. The fewer than 30 annual cases reported by NGOs focusing on non-paediatric patients seem to confirm this imbalance [56]. The impact of this mission-based model is further diminished by the duplication of efforts of multiple teams flying in and out independently needing the process of approval, local coordination, patient selection, etc., carried out in a repetitive fashion [56]. As such, nearly half of the NGOs responding to a survey reported that distance and cost of the trips made it very difficult to increase the activity of their programmes [57]. Therefore, while this first step of CSIA’s undertaking provided facts regarding the under-delivery of CS in LMICs, it also highlighted the need for a focus-shift towards the strengthening of local surgical capacity.

### Cardiac Surgery Intersociety Alliance endorsement principles

In LICs, whether CS is offered at all and at what level largely depends on 4 major determinants: (i) the commitment of governments and their available resources [15]; (ii) the primary healthcare systems, which are often inadequate; (iii) socioeconomic conditions that trigger RHD in the first place and (iv) whether the highly specialized discipline of CS itself is capable of dealing with needs that are very different from those of the industrialized world [3]. While a global collective body of professional cardiac surgical representations cannot influence the socioeconomic circumstances in a country, such an entity’s potential to contribute to the sustainability of local CS through its sheer global presence has proven to be an incentive for governments to committing themselves to this goal [52]. Recent negotiations with the Dutch Government, for instance, regarding a substantial infrastructure and training grant for Mozambique, rest entirely on the presence of the CSIA ‘Seal of Approval’, with its demonstrated and published transparency of processes and with the collective weight of the sponsoring organizations. Similarly, the CSIA requirement for a mentorship agreement with a reasonably high-volume tertiary institution in a middle-income country addresses the fact that in a developing country CS itself differs profoundly from how it is being taught in North America and Europe. This carries Dearani *et al*.’s principle of ‘twinning’ [15, 58], one step further. For an NGO-based model, a symbiotic arrangement between the local champions and a tertiary institution of a HIC has already been crucially augmenting the efficacy of outreach initiatives. In a government-based approach, ‘twinning’ with a functioning institution of a middle-income country has not only the benefit of mirroring the relevant pathologies but also of seeing how resource constraints may be dealt with without jeopardizing service delivery. The CSIA principles expand the twinning to a tri-partite support structure whereby the clinical mentorship comes from a tertiary institution of a middle-income country while research partnerships are encouraged with Universities of industrialized countries.

Finally, the central role of the World Heart Federation in this cardiac surgical initiative has anticipated the necessary cross-fertilization between diagnostic RHD outreach programmes and CS [18]. In a similar multi-country fact-finding study to CSIA’s need-assessment, cardiologists and health economists determined in a Pan African study that costs for valve surgery were below those for primary prevention, greatly refuting the assumption that heart valve surgery is excessively more expensive than current primary prophylaxis—a long-held belief that had contributed widely to de-prioritizing the provision of CS [59].

**Figure 1:**
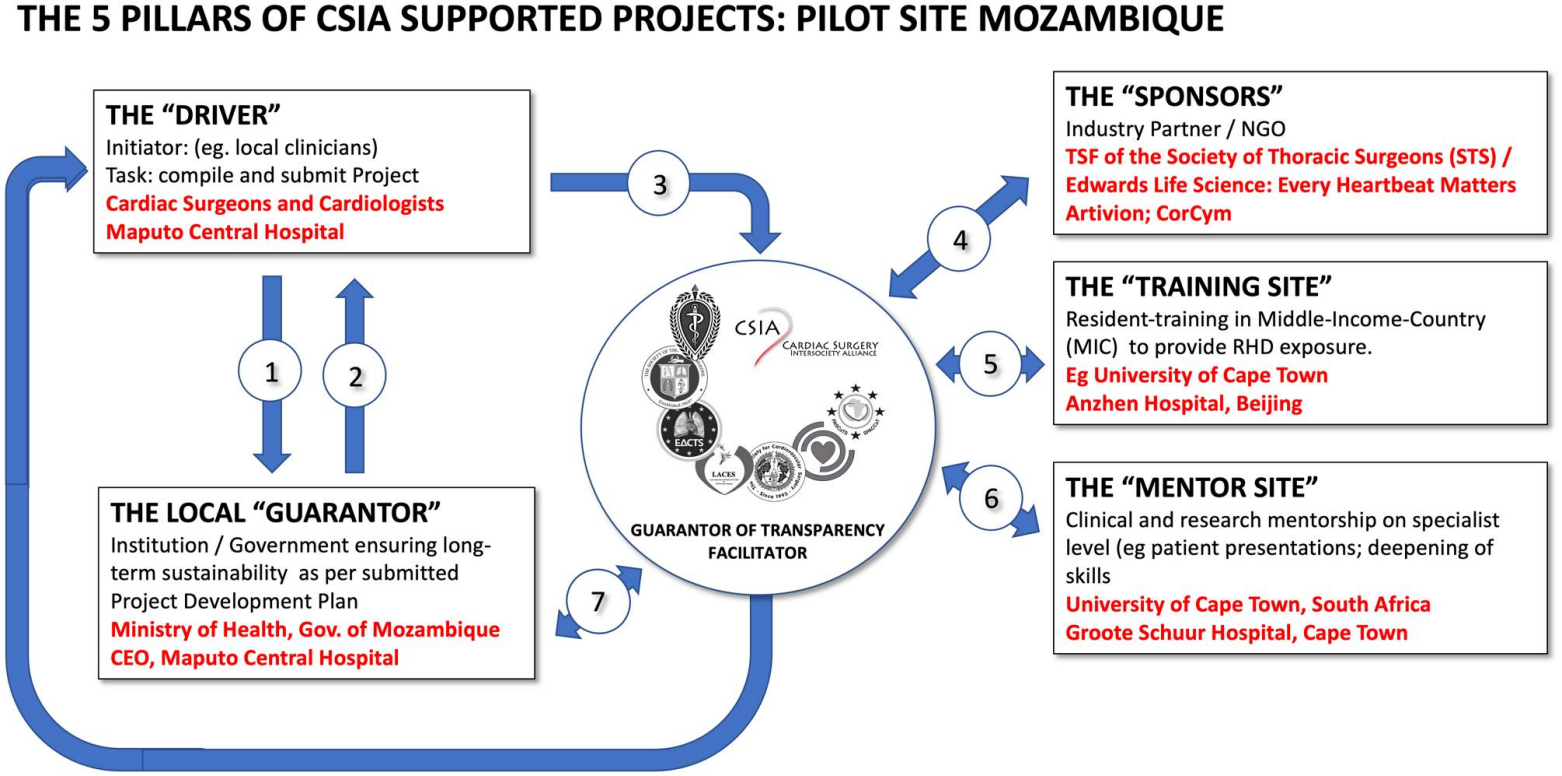
The 7 principles underlying a CSIA agreement as published in the ‘Calls for Proposals to be a Pilot-Site’ in 2019 [50]. Inserted in red are the complying criteria for the Mozambiquean site selection. CSIA: Cardiac Surgery Intersociety Alliance; CEO: chief executive officer; Gov.: government; NGO: non-governmental organization; RHD: rheumatic heart disease; TSF: Thoracic Surgery Foundation

**Figure 2:**
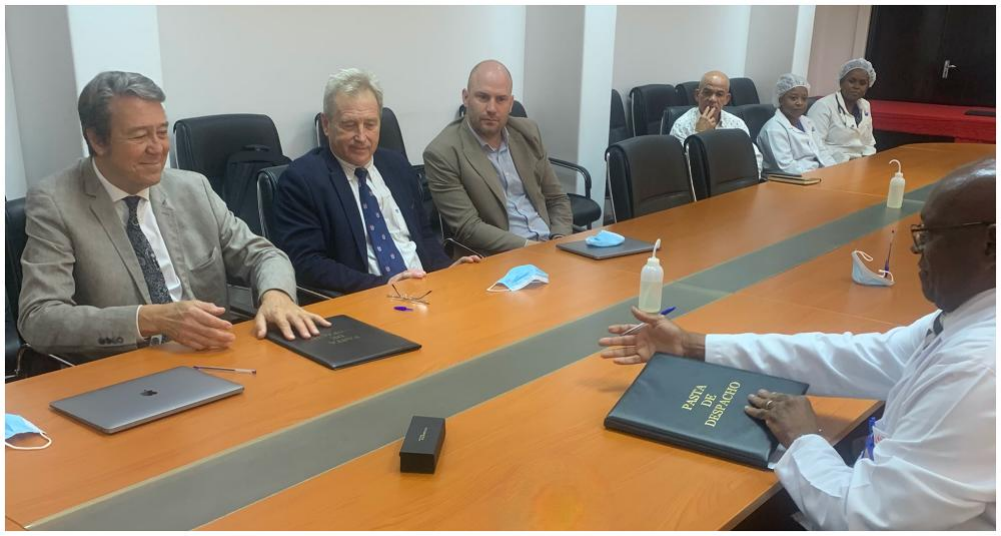
Signing of bilateral Cardiac Surgery Intersociety Alliance Mentorship Agreement between the Mozambiquean Government/Maputo Central Hospital and the University of Cape Town.

Against this background, the criteria for CSIA support were developed [50] with the overriding principle of ‘sustainability of local cardiac surgical capabilities’ in mind (Fig. 1):

- CSIA does not see itself as another NGO. The societies making up the fabric of CSIA largely represent the industrialized world, where not only the financial prowess exists to afford the high level of sophistication that comes with modern cardiovascular medicine but also where most of the leading medical industry resides, as well as those cardiac surgeons who are the backbone of philanthropic initiatives. As such, the unique angle at which CSIA can bring new impetus and energy into the efforts to establish sustainable CS in developing countries lies in ‘focused advocacy’. This advocacy has a good chance of exerting an impact due to the dedicated commitment a ‘united global front of cardiac surgical societies’ can bring to bear.
- CSIA serves as a guarantor of sustainability. The ‘*CSIA Seal of Approval*’ is intended to reassure donors and governments that the outcome of an endorsed programme has a far greater chance of resulting in increased local, sustainable capacity.
- One lesson drawn from the accumulated experience of NGOs relates to local government support. Although many NGO’s intended to create sustainable local capabilities, only a few prevailed [60–62]. Without a full commitment by government—either financially and/or by some integration into the national health and referral system—sustainability is unachievable [6, 7]. This crucial role of government involvement and commitment is demonstrated by both the positive example of the Namibian CS programme [63, 64] as well as the demise of the initial Zimbabwean programme in 2003 [65]. The latter had shown how vulnerable fledgling local programmes are when government support is withdrawn [65]. Informed by these examples, as well as others, ‘one pilar for CSIA endorsement’ is a strong contractual commitment by the respective ministry of health and the CEO of the hospital towards the sustainability of a programme [50] (Fig. 2).
- Addressing very different pathologies under extremely different conditions also means that mentorships by institutions of HICs will not result in the optimal preparation of trainees for the daily demands of their profession at their home countries, such as rheumatic multi-valve surgery or Tb lung surgery. Unintentionally, this training-bias towards degenerative cardiovascular diseases may contribute to the pull-effect of private institutions in LICs, where the rich with their predominantly degenerative pathologies are being treated. Therefore, the ‘second pilar of CSIA support is mentorship’ by a high-volume institution—ideally in a middle-income country where the prevailing pathology and the patient profile mirror those of the supported programme [50].
- As RHD mainly affects the indigent population of LICs, its treatment relies on public health care facilities where staff retention is the greatest hurdle to sustainability. As the strongest pull-factors for healthcare workers from local private facilities and from overseas were found to be non-financial [66], addressing staff frustration in a fledgling cardiac surgical programme is key to long-term stability. As such, advanced training of relevant skills including anaesthesiologists, intensivists and perfusionists, access to enabling technology through advocacy and embedding in a supportive and appreciative professional network of institutions, which share patient profiles and daily challenges were defined as those ‘activities’ through which ‘CSIA can contribute most to the sustainability of cardiac services’ in a developing country.

### Pilot programme selection: circumstances-guided endorsements

In a next step, ‘pilot programs for CSIA support’ were chosen on the basis of the CSIA criteria published in all major cardiothoracic journals in 2019 [50] (Fig. 1). Criteria focused on existing local operating capacity, local championing, governmental and facility support, appropriate identification of a specific gap in care, and the desire to engage in future research. To create public awareness for these criteria, CSIA activities included steering committee meetings, presidential addresses [67, 68] and plenary sessions at annual conferences [69] as well as exhibit hall participation for advertisement and recruitment. For one, only programmes that had succeeded in setting-up a minimum level of independently performed CS were considered in this first round.

System-inherent hurdles typical for LICs were recognized. Generally, these hurdles to the expansion of a programme have a multitude of causes, most prominently the economic realities and resource limitations which serve to restrict access to imported consumables.

The lack of experience in conducting research studies is indirectly also a hurdle to the expansion of a programme. There would certainly be great interest in studies that can address global questions on the basis of the unique patient population of a LIC (e.g. poor INR control in mechanical heart valves) [70]. Properly conducted, such studies would make a struggling programme in a developing country attractive for industry-support. Therefore, the final requirement for CSIA endorsement of an academic mentorship by a renowned tertiary institution [50] will likely be a significant accelerator for the expansion of a programme but will only have a realistic chance of implementation once clinical services have reached a sustainable steady-state.

Following the 2019 call for proposals [50], 11 submissions were received (Table 1). Even though their geographic spectrum reached from Africa to Asia, the vast majority came from Africa [52]. After 3 finalists had been selected, on the basis of fulfilling the CSIA criteria (Fig. 1) closest, site visits were conducted. The 2 selected programmes were Hospital Central Maputo (Mozambique) and King Faisal Hospital Kigali (Rwanda) [52]. Although the 3 years since this endorsement saw the coronavirus disease (COVID) pandemic, much was achieved and most importantly, the ‘CSIA criteria were vindicated’ as they not only proved to be a powerful tool in selecting those programmes that have the best chance of passing the test of the time, but also helped to greatly accelerate their maturation:

**Table 1:** Evolution of CSIA’s role since its inception at the example of Mozambique as one of the 2 selected CSIA pilot sites

Event	Parties involved	Date	Publication
‘CPT Declaration’ calling for the establishment of ‘CSIA’	Participants of 50th anniversary of 1st HTX; Society Presidents; Editors in Chief	December 2017	[1]
Ratification by Societies	STS; AATS; ASCVTS; EACTS; WHF	January 2018 to February 2019	
Need assessment: surgery for RHD	13 LMICs; 3 HICs CSIA 32—author study	August 2018	[2, 3]
‘Call’ for submissions to become a ‘Pilot Site’	Defining 5 pillars of CSIA support	July 2019	[51]
Submissions	(i) FMI Kabul, Afghanistan; (ii) AIIMS Delhi, India; (iii) Tenwek, Kenya; (iv) Univ. Hosp Ilorin, Nigeria (v) Windhoek Central Hospital, Namibia; (iv) Maputo Central, Mozambique; (vii) King Faisal; Kigali, Rwanda; (viii) Tygerberg Hosp, Stellenbosch, South Africa; (ix) Mulago Hospital, Uganda; (x) Univ. Hosp. Lusaka; Zambia; (xi) Pariranyata Hosp, Harare; Zimbabwe	July to September 2019	
Site visits/site selection	King Faisal Hospital, Kigali, Rwanda (selected) Maputo Central Hospital, Mozambique (selected) Parirenyatwa Hospital, Harare, Zimbabwe	February 2020	[52]
‘MV Repair’ training in Cape Town	Senior CT-surgeon from Maputo	March 2020	
COVID pandemic	No surgery	April 2020 to August 2021	
Initiation of ‘Registry for RHD’	UCT—Maputo/Kigali	March 2021	
Intensive hands-on ‘TEE course’	10 members of Maputo team	November 2021	
Participation in ‘UCT’s Journal Club’ and echo teaching programme	Cardiologists and fellows of Maputo	Since February 2022	
‘Refurbishment ICU’ Maputo		February to March 2022	
‘AATS’ Boston ‘presentations’	CT-surgeons from Maputo and Kigali	May 2022	
Monthly ‘Heart Team meetings’ Cape Town-Maputo	Cardiologists, Surgeons, Intensivists	From June 2022	
‘Perfusionist exchange’ Maputo-Kigali plus consumables provided	Senior perfusionist from Maputo	November 2022	
CSIA ‘Presentation ACC/WHF’ New Orleans (virtual)	By senior CT surgeon, Maputo	March 2023	
‘Mentorship Agreement CSIA’ and University of Cape Town (UCT)	Signatories: Department of Health, Eduardo Mondlane Univ., Maputo Central Hospital and UCT	March 2023	
‘1st Mentorship Visit’ of senior ‘UCT Cardiothoracic’ and ‘Anaesthetic’ team	Lined-up difficult cases: surgeons and anaesthetists assisting only	April 2023	
‘1st Mentorship Visit’ of senior ‘UCT Cardiology’ team	Joint patient work-up	April 2023	
‘CSIA presentation’ and ‘AATS’ attendance: Los Angeles	Both senior Cardiothoracic Surgeons from Kigali and Maputo presenting in person	May 2023	
Submission of major ‘CSIA-Dutch Government Grant’	UCT—CSIA (first 2 rounds passed)	May 2023	
Intense ‘Perfusionist training’ at UCT	Perfusionist team from Maputo	June 2023	
‘2nd Mentorship Visit’ of senior ‘UCT Cardiothoracic’, ‘Anaesthetic’ and ‘Intensivist’ team	Lined-up difficult cases: senior surgeons and anaesthetists assisting only; senior intensivist	October 2023	
Training of ‘Intensivists at UCT’	3 senior intensivists from Maputo	November to December 2023	
Training of ‘Cardiologists at UCT’	2 senior cardiologists from Maputo	November to December 2023	
Participation in ‘ICCVA-CASSA Congress’	2 cardiologists of Maputo as speakers	November to December 2023	
‘CT Fellowship’ at ‘UCT’ (4 months)	Senior CT surgery resident from Maputo	April to July 2024	

AATS: American Association for Thoracic Surgery; ACC: American College of Cardiology; AIIMS: All India Institute of Medical Sciences; ASCVTS: Asian Society for Cardiovascular and Thoracic Surgery; COVID: coronavirus disease; CPT: Cape Town; CSIA: Cardiac Surgery Intersociety Alliance; EACTS: The European Association for Cardio-Thoracic Surgery; FMI: French Medical Institute; HIC: high-income country; HTX: Heart transplant; ICCVA-CASSA: International Congress of Cardiothoracic and Vascular Anesthesia-Cardiac Arrhythmia Society of Southern Africa; ICU: Intensive care unit; LMICs: low- to middle-income countries; MV: Mitral valve; RHD: rheumatic heart disease; STS: The Society of Thoracic Surgeons; TEE: Trans-esophageal echocardiography; UCT: University of Cape Town; WHF: World Heart Federation.

- All finalist countries had their ministers of health and the CEO of the applying hospital personally meet the CSIA delegation pledging full support for the programme in writing.
- Subsequent delivery of government/institutional commitments of the chosen programmes towards infrastructure, consumables and capacity allocation surpassed expectations.
- Mentorship agreements with tertiary institutions of middle-income countries with their high incidence of RHD led to tripartite ‘Memoranda of Understanding’ (MoUs) under CSIA auspices providing direct support of the local team in their daily clinical activities and through staff exchanges (Fig. 2).
- Channelling the donation of consumables like heart valves through CSIA rather than their own charitable programmes provided industry with higher visibility while significantly contributing to the increase in case numbers in regions where the high costs of such items is the most limiting factor. Here, too, donations exceeded expectations but so did government responses to match this largesse.
- The fact that funding of staff exchanges and teaching programmes through society foundations such as Thoracic Surgery Foundation (TSF) or the American Association for Thoracic Surgery (AATS) Foundation was denominated in US Dollars had an additional multiplier effect against the depreciating currencies of developing countries

## PROGRESS REPORT

Given the legitimate prudence of the societies towards entering the parquet of health policy in developing countries and also towards the hitherto untested structure of an intersociety alliance the in-depth multi-society review—overlapping with the pandemic—dedicated the initial years to getting all framework conditions resolved.

**Figure 3:**
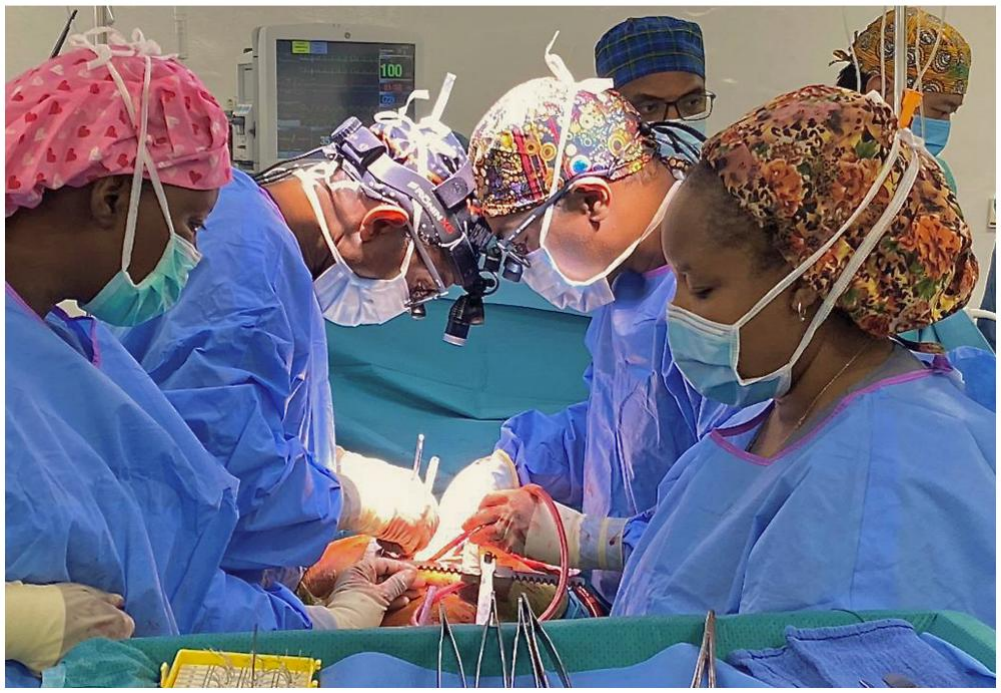
Mentored cardiac surgery performed in Rwanda by Rwandan team.

**Figure 4:**
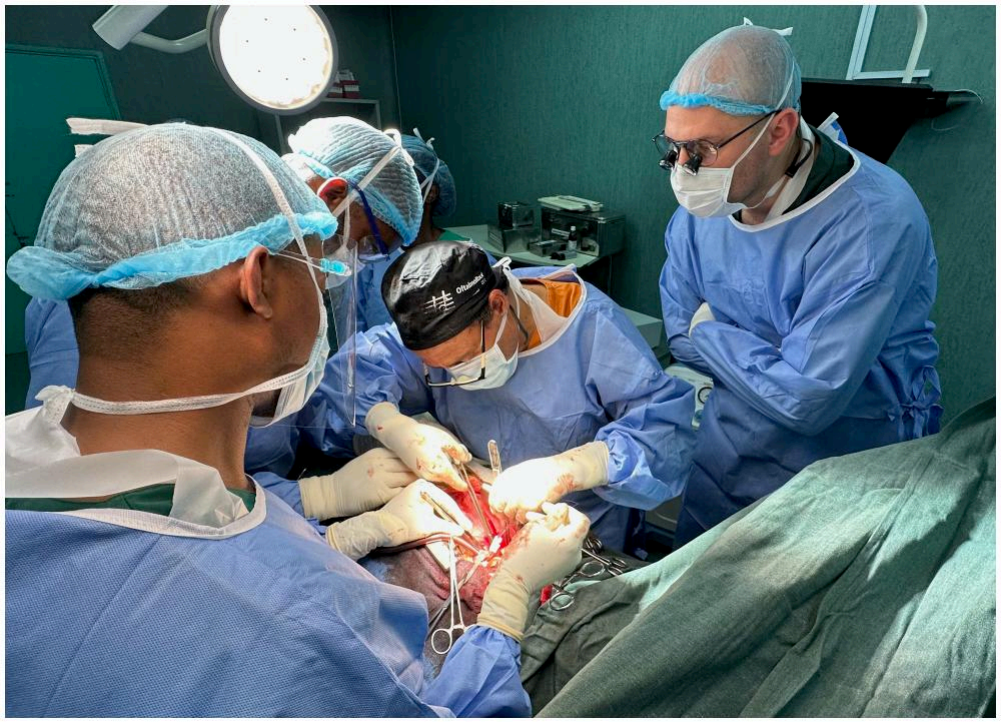
Mentorship visit by team of the University of Cape Town at Maputo Central Hospital, Eduardo Mondlane University, Mozambique. While scrubbed in, the University of Cape Town team strictly observed the principle of the local team performing the operation.

Donated equipment, supplies and mentorship efforts have been made possible by the TSF (the charitable arm of the Society of Thoracic Surgeons) and Edwards Lifesciences through their ‘Every Heartbeat Matters’ philanthropic programme. TSF provided the seed-funds for CSIA activities. Significantly, equally in October 2022, ‘100 heart valves’ were donated to CSIA—50 by Artivion Inc, directed to the programme in Mozambique, and 50 by CorCym, directed to Rwanda. As had been desired, these critical donations were the basis for the Government to permit a significant ‘increase in the number of performed operations’. As the emphasis of CSIA support is on the strengthening of local cardiac surgical capacity, the principle of surgery being conducted by local surgeons and their teams was upheld throughout (Figs 3 and 4).

‘Mozambique’ is one of the poorest countries in the world, ranking 181 out of 189 countries on the human development index. As part of the CSIA initiative:

- Being a middle-income institution, the UCT/Groote Schuur Hospital took on ‘mentorship’ for Maputo Central Hospital/Eduardo Mondlane University (Fig. 1, Table 1).
- ‘Resident training’ at high-volume Institutions in India and China are being initiated. A Fellowship for a senior resident of the cardiac surgical team of Maputo Central has been arranged at UCT for 2024.
- Staff ‘training at tertiary institution with significant share of RHD’ have been a major focus of CSIA’s role in Mozambique (see Table 1). Starting in June 2023, the teams in Cape Town and Maputo have also instituted monthly video conferences in which challenging surgical ‘cases are presented’ and discussed among the heart teams of both institutions. ‘Telephonic direct lines for intraoperative advice’ have equally been established and utilized. As part of the agreement with the minister of health, CSIA efforts were ‘matched by the government’ by the purchase of a ‘second heart lung machine, 120 additional oxygenators’ and 20 ‘additional mechanical valve prostheses’. A ‘state-of-the-art modern cardiac’ intensive care unit (ICU) is currently being built. Since then, intra-bypass anaesthetics could be switched to from iv to isoflurane on the new heart lung machine for the training of which the ‘perfusionist’ team from Mozambique spent time in Cape Town. Further extended training visits of Mozambiquean specialist and support staff at UCT and mentoring visits of Cardiothoracic, Anaesthetic, Cardiology and Intensivist teams from UCT have become increasingly frequent.
- Overall, CSIA’s involvement in Mozambique has had the effect not only of ‘qualitatively’ strengthening the CS services of the programme, but also ‘quantitatively’ as the Government committed itself to almost ‘doubling the annual case volume’ over the next 2 years. Post-COVID case numbers slowly recovered from 10 in 2021 to 35 in 2022 to 43 in 2023 (including double and triple-valve surgeries) restricted by 1 ICU bed per week. Two ICU beds/week plus the intense staff and support-staff training of the past 18 months promise a noticeable increase of open-heart cases over the next 2 years.

‘Rwanda’: King Faisal Hospital in Kigali, Rwanda, is the site of the open-heart surgery programme in Rwanda. An NGO, Team Heart, had obtained CSIA’s ‘seal of approval’ for Rwanda as a CSIA pilot site after it had driven the development of local CS capacity in Rwanda for several years. While a young Rwandan surgeon completed his ‘general- and cardiothoracic surgery training in South Africa’, expatriate teams from Australia, Belgium, USA and Canada enabled CS in more than 500 adult and paediatric patients. After passing the South African board exams, the local surgeon returned to Rwanda in 2019 with Team Heart taking on the mentorship role. COVID interrupted the efforts to establish sustainable CS at King Faisal, too. Since then, ‘Team Heart’ has completed 5 surgical mentorship trips with the entire ‘focus’ on preparing the local team to ‘perform independent cardiac surgical procedures’. By early 2023, the local heart team at the King Faisal Hospital had crossed the threshold to the performance of independent cardiac surgical procedures, allowing future surgical mentorship trips to ‘prepare’ them for performing ‘operations of increasing complexity’. Strengthening the link between the 2 sites, a ‘perfusionist’ of the ‘Mozambiquean’ team was invited to travel to Kigali and to participate in the ‘Team Heart’ mentorship experience. Upon his return to Mozambique, ‘Team Heart donated further heart valves’ for the Mozambiquean programme, as well as other equipment enabling the Maputo programme to re-initiate their open-heart surgery programme, while they awaited the arrival of the heart valves donated through CSIA.

## CONCLUDING CARDIAC SURGERY INTERSOCIETY ALLIANCE’S INITIAL YEARS OF ACTIVITY

**Figure 5:**
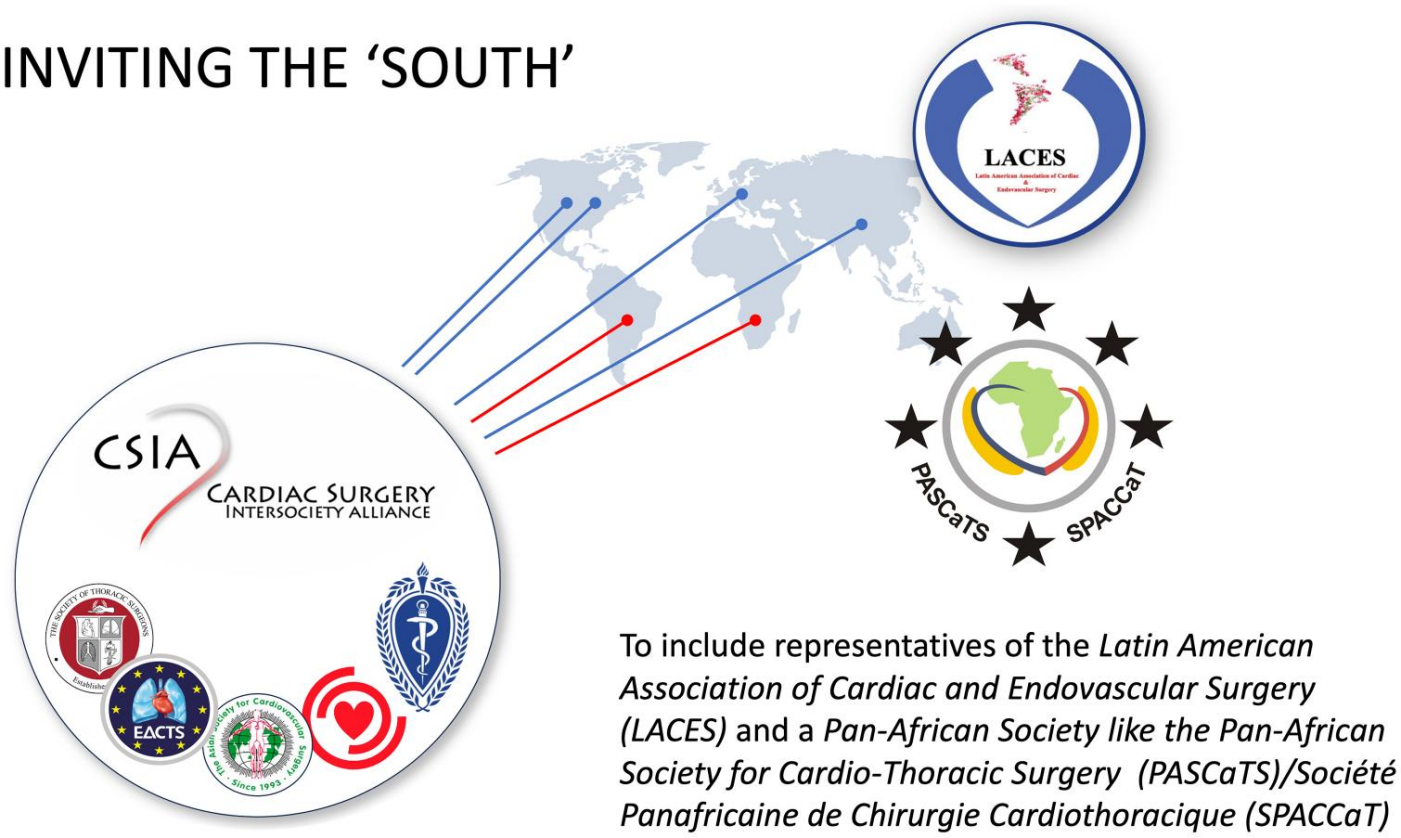
When CSIA was established in 2018, neither Latin America nor Africa had fully fledged continent-spanning Cardiac Surgical Societies. As from 2023 CSIA lives up to a global claim by having admitted Latin American Association of Cardiac- and Endovascular Surgery and Pan African Societies for Cardiothoracic Surgery at its meeting during the annual European Association for Cardio-Thoracic Surgery congress in Vienna.

A unique umbrella structure comprising the Society of Thoracic Surgeons (STS), the American Association for Thoracic Surgery (AATS), the Asian Society for Cardiovascular and Thoracic Surgery (ASCVTS), the European Association for Cardio-Thoracic Surgery (EACTS) and the World Heart Federation (WHF) has been created and ratified by all participating societies, whereby each Society appoints 2 representatives and collectively may invite additional ‘at large’ members to serve on the steering committee. Usually, CSIA delegates have been involved in outreach activities to developing countries before. The administrative seat of CSIA is the ‘University of Cape Town’. At the steering committee’s meeting at the 2023 EACTS meeting in Vienna, the Latin American Association of Cardiac- and Endovascular Surgery (LACES) and Pan African Societies for Cardiothoracic Surgery (PASCaTS) were admitted to the CSIA umbrella to include cardiac surgical representations of the Southern Hemisphere (Fig. 5).

- CSIA, created as a call-to-arms for leaders of CS, cardiology, government and the medical device industry to pool their efforts to increase access to life-saving CS in the developing world, has succeeded in identifying and helping to resource the ‘first two pilot programmes’ for increasing CS in the developing world. This achievement has been realized despite all the impediments posed by the COVID pandemic.
- The initial 5 years vindicated the core principle on which the CSIA concept rests: the ‘power of advocacy’ by an organization that represents the weight of all major cardiac societies. ‘Governments feel supported’ in the daunting task of establishing one of the most technologically demanding surgical disciplines. ‘Donors feel reassured’ that structured oversight and a clear end-point guarantee the most effective use of their contributions. ‘Staff morale’ in mostly isolated out-post programmes gets significantly boosted by feeling embedded in the global cardiac community, thereby significantly lowering the threshold for staff retention.
- Both pilot programmes have ‘demonstrated measurable progress’ in performing ‘independent cardiac surgical procedures’. Support for the sites from hospital and government is highly encouraging: In Mozambique, the Ministry of Health has contributed a 2nd heart lung machine and—in addition to the government-sustained basic CS programme—oxygenators and heart valve prostheses and a state-of-the-art cardiac ICU in order to support the continued performance of open-heart operations. In Rwanda, the King Faisal Hospital has committed to the purchase of all supplies needed for CS, and, in addition, in September, 2023, is renovating an operating theatre for CS. Supply chain issues have also been successfully addressed at both sites.

## WAY FORWARD

CSIA has proven to be a ‘powerful tool’ for stream-lining the often widely scattered efforts towards sustainable cardiac surgical services in developing countries. CSIA is ‘not another NGO’ and as such its value lies in ‘focusing and augmenting existing initiatives’ through its clear principles and well-defined end-point. Its involvement in 2 concrete projects in Africa tested the underlying assumption of the facilitating power of a united professional front in all key components of the journey of a fledgling programme towards sustainability from obtaining government commitments to opening avenues to industry and granting agencies.

As the undeniable and massive need for CS for the forgotten millions in LIC’s around the globe only increases with each passing day, the next task will be to invite the ‘local stake holders to signing on to the CSIA concept’. On the one hand, the examples of Namibia and Uganda have shown how far the ‘re-positioning of the governments’ of developing countries towards the recognition of the national economics of CS evolved. As these patients are in the prime of their lives, working, raising families and potentially contributing to the tax base, they represent an enormous lost economic resource for affected countries. On the other hand, ‘industry’ has shown that it is prepared to step up their efforts if their ‘contribution’ is not diluted through multiple often redundant independent initiatives but ‘enhanced by an overlying principle’ that promises the possibility of an albeit modest market entry as the end-point. Increasingly, a 3rd player emerged in form of ‘regional societies and academic initiatives’ [63, 64, 71–73], which will be important to be won over to the CSIA concept.

There is still a research gap for CS in clinical RHD in LICs. Both basic research [74, 75] as well as non-interventional clinical studies [27, 76] into RHD have taken off in previous years. In the near absence of CS, however, and no comparable international collaborations in place which cardiology established a few years ago [75, 77], the sky is the limit for highly targeted clinical research focusing on rheumatic heart valve surgery under the circumstances of LICs.

There is also a sizable pool of graduate students who always knew that they want to dedicate their careers to surgery in LICs but would not know how to get involved. The publication record of PhD students involved in the first 5 years of CSIA [36, 52, 78] is impressive and should serve as an encouragement for others.

**Figure 6:**
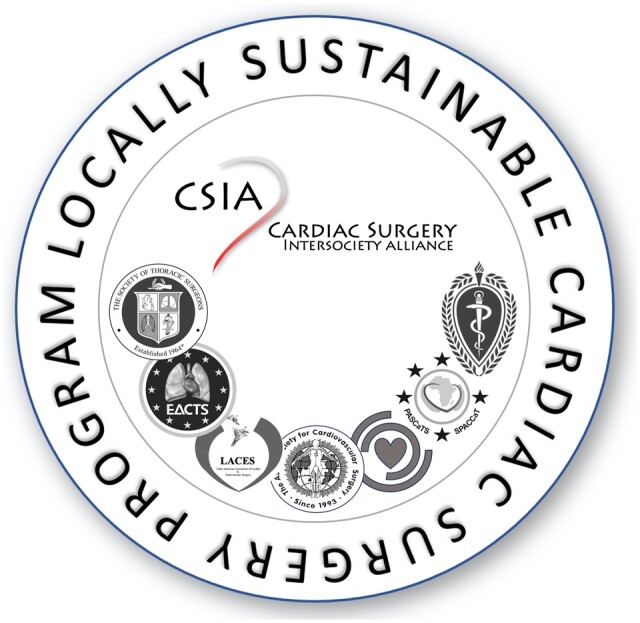
The CSIA ‘Seal of Approval’ represents the underlying principle of the alliance: the adherence of a programme to the principle of local capacity-building and the commitment to focus on rheumatic heart disease in accordance with its incidence. CSIA: Cardiac Surgery Intersociety Alliance.

With the CSIA structures being consolidated and the underlying principle vindicated in 2 pilot programmes, time seems to be ripe to take the next steps. The primary goals for the immediate future must therefore be:

- To proceed from pilot programmes to the wider adaptation of a ‘CSIA Seal of Approval’ (Fig. 6), whose fundamental underlying principle must be an unqualified commitment to the establishment or strengthening of local cardiac surgical capacity along the principles published in 2019 [50]. The spectrum of consignees of this ‘Seal of Approval’ can stretch from Government programmes to NGOs. For the time being, RHD will remain the sole focus of CSIA.
- To invite ‘key players of regional’ societies, academic initiatives and ‘global health structures’ to get actively involved in the steering committee of CSIA.
- To ‘ingrain’ the belief and enthusiasm for the CSIA concept even deeper in the ‘DNA of the entire membership of the societies’ through internal advocacy.

Applying measurable metrics [15] to the success of the CSIA will augment its momentum in the cardiac community. Growing specialist- and support-staff-skills will be as central as increasing case numbers. Key goal will have to remain the establishment of a team of local experts who cover a spectrum of ‘skills’ that not only guarantees a stable modern cardiothoracic surgical programme treating RHD but that eventually may become the foundation for also tackling the most common congenital heart defects on the basis of local capabilities [79].

‘If not this, what? If not here, where? If not now, when? If not us, who?’

**Conflict of interest:** none declared.

## ABBREVIATIONS

AATS: American Association for Thoracic Surgery
ASCVTS: Asian Society for Cardiovascular and Thoracic Surgery
COVID: Coronavirus disease
CS: Cardiac surgery
CSIA: Cardiac Surgery Intersociety Alliance
CTD: Cape Town Declaration
EACTS: The European Association for Cardio-Thoracic Surgery
HICs: High-income countries
ICU: Intensive care unit
LACES: Latin American Association of Cardiac and Endovascular Surgery
LICs: Low-income countries
LMICs: Low- to middle-income countries
NGOs: Non-governmental Organizations
PASCaTS: Pan African Society for Cardiothoracic Surgery
RHD: Rheumatic heart disease
STS: The Society of Thoracic Surgeons
TSF: Thoracic Surgery Foundation, the charitable arm of STS
UCT: University of Cape Town
WHF: World Heart Federation

## References

[1] Zilla P , Bolman RM, Yacoub MH, Beyersdorf F, Sliwa K, Zuhlke L et al The Cape Town Declaration on access to cardiac surgery in the developing world. J Thorac Cardiovasc Surg 2018;156:2206–9. 30082076 10.1016/j.jtcvs.2018.06.002

[2] Zilla P , Yacoub M, Zuhlke L, Beyersdorf F, Sliwa K, Khubulava G et al Global unmet needs in cardiac surgery. Glob Heart 2018;13:293–303. 30245177 10.1016/j.gheart.2018.08.002

[3] Zilla P , Bolman RM 3rd, Boateng P, Sliwa K. A glimpse of hope: cardiac surgery in low- and middle-income countries (LMICs). Cardiovasc Diagn Ther 2020;10:336–49. 32420116 10.21037/cdt.2019.11.03 PMC7225428

[4] Yankah C , Fynn-Thompson F, Antunes M, Edwin F, Yuko-Jowi C, Mendis S et al Cardiac surgery capacity in sub-saharan Africa: quo vadis? Thorac Cardiovasc Surg 2014;62:393–401. 24955755 10.1055/s-0034-1383723

[5] Vervoort D , Swain JD, Pezzella AT, Kpodonu J. Cardiac surgery in low- and middle-income countries: a state-of-the-art review. Ann Thorac Surg 2021;111:1394–400. 32771467 10.1016/j.athoracsur.2020.05.181

[6] Nwafor IA , Vickram A, Osenmobor KO. Surgical ‘Safari’ vs. educational program: experience with international cardiac surgery missions in Nigeria. Braz J Cardiovasc Surg 2020;35:918–26. 33306318 10.21470/1678-9741-2020-0155 PMC7731857

[7] Tefera E , Nega B, Yadeta D, Chanie Y. Humanitarian cardiology and cardiac surgery in sub-saharan Africa: can we reshape the model? World J Pediatr Congenit Heart Surg 2016;7:727–31. 27834766 10.1177/2150135116668834

[8] Mohd Zain MR , Shamsuddin AM, Mamat AZ, Mokhtar AM, Ali S, Chen YC et al Humanitarian mission in pediatric cardiothoracic surgery: a recipient’s perspective. Front Pediatr 2019;7:230. 31231625 10.3389/fped.2019.00230 PMC6568032

[9] Commerford PJ. Valvular heart disease in South Africa in 2005. S Afr Med J 2005;95:568, 570, 572–4. 16200999

[10] Mayosi B , Robertson K, Volmink J, Adebo W, Akinyore K, Amoah A et al The Drakensberg declaration on the control of rheumatic fever and rheumatic heart disease in Africa. S Afr Med J 2006;96(3 Pt 2):246. 16610104

[11] Remenyi B , Carapetis J, Wyber R, Taubert K, Mayosi BM, World HF; World Heart Federation. Position statement of the World Heart Federation on the prevention and control of rheumatic heart disease. Nat Rev Cardiol 2013;10:284–92. 23546444 10.1038/nrcardio.2013.34

[12] Beyer DC , Mohideen N. The role of physicians and medical organizations in the development, analysis, and implementation of health care policy. Semin Radiat Oncol 2008;18:186–93. 18513628 10.1016/j.semradonc.2008.01.006

[13] Thakur N , McGarry ME, Oh SS, Galanter JM, Finn PW, Burchard EG; ATS Health Equality Committee. The lung corps’ approach to reducing health disparities in respiratory disease. Ann Am Thorac Soc 2014;11:655–60. 24697756 10.1513/AnnalsATS.201402-061AR PMC4225795

[14] Celedon JC , Roman J, Schraufnagel DE, Thomas A, Samet J. Respiratory health equality in the United States. The American thoracic society perspective. Ann Am Thorac Soc 2014;11:473–9. 24625275 10.1513/AnnalsATS.201402-059PS PMC4225793

[15] Dearani JA , Jacobs JP, Bolman RM 3rd, Swain JD, Vricella LA, Weinstein S et al Humanitarian outreach in cardiothoracic surgery: from setup to sustainability. Ann Thorac Surg 2016;102:1004–11. 27319988 10.1016/j.athoracsur.2016.03.062

[16] Zühlke LJ , Beaton A, Engel ME, Hugo-Hamman CT, Karthikeyan G, Katzenellenbogen JM et al Group A Streptococcus, acute rheumatic fever and rheumatic heart disease: epidemiology and clinical considerations. Curr Treat Options Cardiovasc Med 2017;19:15. 28285457 10.1007/s11936-017-0513-y PMC5346434

[17] Tretter JT , Jacobs JP. Global leadership in paediatric and congenital cardiac care: “global health advocacy, lift as you rise—an interview with Liesl J. Zuhlke, MBChB, MPH, PhD”. Cardiol Young 2021;31:1549–56. 34602114 10.1017/S104795112100411X

[18] Sliwa K , White A, Milan P, Olga Mocumbi A, Zilla P, Wood D. Momentum builds for a global response to rheumatic heart disease. Eur Heart J 2018;39:4229–32. 30576470 10.1093/eurheartj/ehy763

[19] Watkins DA , Beaton AZ, Carapetis JR, Karthikeyan G, Mayosi BM, Wyber R et al Rheumatic heart disease worldwide: JACC Scientific Expert Panel. J Am Coll Cardiol 2018;72:1397–416. 30213333 10.1016/j.jacc.2018.06.063

[20] Institute for Health Metrics and Evaluation. Global Burden of Diseases. 2023. https://www.healthdata.org/data-tools-practices/interactive-visuals/gbd-compare (30 June 2023, date last accessed).

[21] Salie MT , Yang J, Ramirez Medina CR, Zuhlke LJ, Chishala C, Ntsekhe M et al; RHDGen Network Consortium. Data-independent acquisition mass spectrometry in severe rheumatic heart disease (RHD) identifies a proteomic signature showing ongoing inflammation and effectively classifying RHD cases. Clin Proteomics 2022;19:7. 35317720 10.1186/s12014-022-09345-1 PMC8939134

[22] World-Health-Organisation. Rheumatic fever and rheumatic heart disease. WHO Resolution WHA71.14. Seventy-first World Health Assembly 26 May 2018, 2018.

[23] Rwebembera J , Nascimento BR, Minja NW, de Loizaga S, Aliku T, Dos Santos LPA et al Recent advances in the rheumatic fever and rheumatic heart disease continuum. Pathogens 2022;11:179. 35215123 10.3390/pathogens11020179 PMC8878614

[24] Beaton A , Okello E, Rwebembera J, Grobler A, Engelman D, Alepere J et al Secondary antibiotic prophylaxis for latent rheumatic heart disease. N Engl J Med 2022;386:230–40. 34767321 10.1056/NEJMoa2102074

[25] Rwebembera J , Marangou J, Mwita JC, Mocumbi AO, Mota C, Okello E et al 2023 World Heart Federation guidelines for the echocardiographic diagnosis of rheumatic heart disease. Nat Rev Cardiol 2024;21:250–63. doi: 10.1038/s41569-023-00940-9. 37914787

[26] Okello E , Longenecker CT, Beaton A, Kamya MR, Lwabi P. Rheumatic heart disease in Uganda: predictors of morbidity and mortality one year after presentation. BMC Cardiovasc Disord 2017;17:20. 28061759 10.1186/s12872-016-0451-8 PMC5219796

[27] Beaton A , Okello E, Rwebembera J, Grobler A, Engelman D, Alepere J et al Refining risk stratification among children with latent rheumatic heart disease. Circulation 2023;147:1848–50. 37307310 10.1161/CIRCULATIONAHA.122.063194

[28] Verral A. Funding for vaccine development to help prevent rheumatic fever. Official Website of the New Zealand Government (Beehivegovtnz). 2021. https://www.beehive.govt.nz/release/funding-vaccine-development-help-prevent-rheumatic-fever (30 June 2023, date last accessed).

[29] Bukhman G , Mocumbi AO, Gupta N, Amuyunzu-Nyamongo M, Echodu M, Gomanju A et al; NCDI Poverty Network. From a Lancet Commission to the NCDI Poverty Network: reaching the poorest billion through integration science. Lancet 2021;398:2217–20. 34687660 10.1016/S0140-6736(21)02321-7

[30] Karthikeyan G , Connolly SJ, Ntsekhe M, Benz A, Rangarajan S, Lewis G et al; INVICTUS Investigators. The INVICTUS rheumatic heart disease research program: rationale, design and baseline characteristics of a randomized trial of rivaroxaban compared to vitamin K antagonists in rheumatic valvular disease and atrial fibrillation. Am Heart J 2020;225:69–77. 32474206 10.1016/j.ahj.2020.03.018

[31] Vervoort D , Yilgwan CS, Ansong A, Baumgartner JN, Bansal G, Bukhman G et al Tertiary prevention and treatment of rheumatic heart disease: a National Heart, Lung, and Blood Institute working group summary. BMJ Glob Health 2023;8:e012355. 10.1136/bmjgh-2023-012355 PMC10619050 37914182

[32] Watkins DA , Johnson CO, Colquhoun SM, Karthikeyan G, Beaton A, Bukhman G et al Global, regional, and national burden of rheumatic heart disease, 1990-2015. N Engl J Med 2017;377:713–22. 28834488 10.1056/NEJMoa1603693

[33] Passos LSA , Nunes MCP, Zilla P, Yacoub MH, Aikawa E. Raising awareness for rheumatic mitral valve disease. Glob Cardiol Sci Pract 2020;2020:e202026. 33426043 10.21542/gcsp.2020.26 PMC7768627

[34] Simpson MT , Kachel M, Neely RC, Erwin WC, Yasin A, Patel A et al Rheumatic heart disease in the developing world. Struct Heart 2023;7:100219. 38046860 10.1016/j.shj.2023.100219 PMC10692356

[35] Marijon E , Ou P, Celermajer DS, Ferreira B, Mocumbi AO, Jani D et al Prevalence of rheumatic heart disease detected by echocardiographic screening. N Engl J Med 2007;357:470–6. 17671255 10.1056/NEJMoa065085

[36] Enumah ZO , Bolman RM 2nd. Cardiac surgery in low- and middle-income countries: can we move the needle? Ann Thorac Surg 2021;111:1400–1. 32961140 10.1016/j.athoracsur.2020.06.132

[37] Acheampong B , Parra DA, Aliyu MH, Moon TD, Soslow JH. Smartphone interfaced handheld echocardiography for focused assessment of ventricular function and structure in children: a pilot study. Echocardiography 2020;37:96–103. 31879998 10.1111/echo.14575 PMC7067587

[38] Afifi A , Hosny H, Yacoub M. Rheumatic aortic valve disease—when and who to repair? Ann Cardiothorac Surg 2019;8:383–9. 31240182 10.21037/acs.2019.05.01 PMC6562081

[39] Chotivatanapong T. Rheumatic mitral valve repair: a personal perspective and results. Asian Cardiovasc Thorac Ann 2020;28:366–70. 32436717 10.1177/0218492320927315

[40] Ramakumar V , Benz AP, Karthikeyan G. Long-term oral anticoagulation for atrial fibrillation in low and middle income countries. Indian Heart J 2021;73:244–8. 33865530 10.1016/j.ihj.2021.02.003 PMC8065364

[41] Appa H , Park K, Bezuidenhout D, van Breda B, de Jongh B, de Villiers J et al The technological basis of a balloon-expandable TAVR system: non-occlusive deployment, anchorage in the absence of calcification and polymer leaflets. Front Cardiovasc Med 2022;9:791949. 35310972 10.3389/fcvm.2022.791949 PMC8928444

[42] Zilla P , Williams DF, Bezuidenhout D. TAVR for patients with rheumatic heart disease: opening the door for the many? J Am Coll Cardiol 2021;77:1714–6. 33832597 10.1016/j.jacc.2021.02.044

[43] Webb A , Obungoloch J. Five steps to make MRI scanners more affordable to the world. Nature 2023;615:391–3. 36918678 10.1038/d41586-023-00759-x

[44] Zilla P , Bolman RM, Yacoub MH, Beyersdorf F, Sliwa K, Zuhlke L et al The Cape Town declaration on access to cardiac surgery in the developing world. S Afr Med J 2018;108:702–4. 30182888 10.7196/SAMJ.2018.v108i9.13102

[45] Zilla P , Bolman RM, Yacoub MH, Beyersdorf F, Sliwa K, Zuhlke L et al The Cape Town declaration on access to cardiac surgery in the developing world. Eur J Cardiothorac Surg 2018;54:407–10. 30113630 10.1093/ejcts/ezy272

[46] Zilla P , Bolman RM, Yacoub MH, Beyersdorf F, Sliwa K, Zuhlke L et al The Cape Town Declaration on access to cardiac surgery in the developing world. Asian Cardiovasc Thorac Ann 2018;26:535–9. 30099880 10.1177/0218492318791359

[47] Zilla P , Bolman RM, Yacoub MH, Beyersdorf F, Sliwa K, Zuhlke L et al The Cape Town Declaration on access to cardiac surgery in the developing world. Ann Thorac Surg 2018;106:930–3. 30082030 10.1016/j.athoracsur.2018.05.020

[48] Zilla P , Bolman RM, Yacoub MH, Beyersdorf F, Sliwa K, Zuhlke L et al The Cape Town Declaration on access to cardiac surgery in the developing world. Cardiovasc J Afr 2018;29:256–9. 30080213 10.5830/CVJA-2018-046 PMC6291809

[49] Zilla P , Zuhlke L, Sliwa K, Commerford P. The African context of the Cape Town Declaration. Cardiovasc J Afr 2018;29:204. 30204217 PMC6421547

[50] CSIA. Call for proposals to be a pilot site for CSIA supported programs. Eur J Cardiothorac Surgery 2019;56:425–6.

[51] Boateng P , Bolman RM 3rd, Zilla P; CSIA. Cardiac surgery for the forgotten millions: the way forward. Cardiac Surgery Intersociety Alliance (CSIA) Site Selection Criteria. J Thorac Cardiovasc Surg 2019;158:818–9. 31324422 10.1016/j.jtcvs.2019.06.030

[52] Enumah ZO , Bolman RM, Zilla P, Boateng P, Wilson B, Kumar AS et al United in earnest: first pilot sites for increased surgical capacity for rheumatic heart disease announced by cardiac surgery intersociety alliance. Ann Thorac Surg 2021;111:1931–6. 33840453 10.1016/j.athoracsur.2020.11.043

[53] Sliwa K , Zilla P. 50th anniversary of the first human heart transplant-How is it seen today? Eur Heart J 2017;38:3402–4. 29232446 10.1093/eurheartj/ehx695

[54] Casey KM. The global impact of surgical volunteerism. Surg Clin North Am 2007;87:949–60, ix. 17888791 10.1016/j.suc.2007.07.018

[55] Cardarelli M , Vaikunth S, Mills K, DiSessa T, Molloy F, Sauter E et al Cost-effectiveness of humanitarian pediatric cardiac surgery programs in low- and middle-income countries. JAMA Netw Open 2018;1:e184707. 30646368 10.1001/jamanetworkopen.2018.4707 PMC6324367

[56] Vervoort D , Guetter CR, Munyaneza F, Trager LE, Argaw ST, Abraham PJ et al Non-governmental organizations delivering global cardiac surgical care: a quantitative impact assessment. Semin Thorac Cardiovasc Surg 2022;34:1160–5. 34407434 10.1053/j.semtcvs.2021.08.010

[57] Nguyen N , Jacobs JP, Dearani JA, Weinstein S, Novick WM, Jacobs ML et al Survey of nongovernmental organizations providing pediatric cardiovascular care in low- and middle-income countries. World J Pediatr Congenit Heart Surg 2014;5:248–55. 24668973 10.1177/2150135113514458 PMC4276142

[58] Dearani JA , Neirotti R, Kohnke EJ, Sinha KK, Cabalka AK, Barnes RD et al Improving pediatric cardiac surgical care in developing countries: matching resources to needs. Semin Thorac Cardiovasc Surg Pediatr Card Surg Annu 2010;13:35–43. 20307859 10.1053/j.pcsu.2010.02.001

[59] Coates MM , Sliwa K, Watkins DA, Zuhlke L, Perel P, Berteletti F et al An investment case for the prevention and management of rheumatic heart disease in the African Union 2021-30: a modelling study. Lancet Glob Health 2021;9:e957–e66. 33984296 10.1016/S2214-109X(21)00199-6 PMC9087136

[60] Van HD. Nineteen years of single institute experiences with Sorin Bicarbon prosthesis. Ann Thorac Cardiovasc Surg 2019;25:192–9. 30867384 10.5761/atcs.oa.18-00223 PMC6698711

[61] Scholtz A. Professor Sir Magdi Yacoub and the Aswan Heart Centre. J Am Coll Cardiol 2018;72:1417–21. 30213334 10.1016/j.jacc.2018.08.005

[62] Yacoub M , Labib D. Toward meeting the challenges of improving cardiovascular health in Egypt. Circulation 2021;143:1341–2. 33819079 10.1161/CIRCULATIONAHA.119.040939

[63] Forcillo J , Watkins DA, Brooks A, Hugo-Hamman C, Chikoya L, Oketcho M et al; Contributors from Namibia, Zambia, and Uganda. Making cardiac surgery feasible in African countries: experience from Namibia, Uganda, and Zambia. J Thorac Cardiovasc Surg 2019;158:1384–93. 30819574 10.1016/j.jtcvs.2019.01.054

[64] Shidhika F , Mureko A, Feris N, de Mey W, Du Toit H, Beshir S et al Cardiac catheterization and surgery in Namibia. SA Heart 2020;17:14–8.

[65] Machawira SP , Muteweye W, Mutetwa E, Kajese S. Towards sustainable open heart surgery in Zimbabwe. Front Pediatr 2022;10:806411. 35865707 10.3389/fped.2022.806411 PMC9294396

[66] Weldegebriel Z , Ejigu Y, Weldegebreal F, Woldie M. Motivation of health workers and associated factors in public hospitals of West Amhara, Northwest Ethiopia. Patient Prefer Adherence 2016;10:159–69. 26929608 10.2147/PPA.S90323 PMC4760664

[67] de Paulis R. Presidential address: “Our House of Glass”. 33rd annual meeting of the European Association for Cardiothoracic Surgery (EACTS), Lisbon October 3–5, 2019. 2019.

[68] Beyersdorf F. Presidential address: “Innovation and Transformation”. 36th annual meeting of the European Association for Cardiothoracic Surgery, Milan, October 5–8, 2022, 2022.

[69] Bolman RM. Plenary Session. 99th annual meeting of the American Association for Thoracic Surgery (AATS), Toronto, May 4–7, 2019, 2019.

[70] Zilla P, Human P, Pennel T Mechanical valve replacement for patients with rheumatic heart disease: the reality of INR control in Africa and beyond. Front Cardiovasc Med 2024; 11:1347838. doi: 10.3389/fcvm2024.1347838. PMC10884232 38404722

[71] Nwiloh J. Establishment of an African regional cardiothoracic surgery database. Niger J Cardiovasc Thorac Surg 2020;5:1–2.

[72] Edwin F , Tettey M, Aniteye E, Tamatey M, Sereboe L, Entsua-Mensah K et al The development of cardiac surgery in West Africa—the case of Ghana. Pan Afr Med J 2011;9:15. 22355425 10.4314/pamj.v9i1.71190 PMC3215537

[73] Aliku TO , Lubega S, Namuyonga J, Mwambu T, Oketcho M, Omagino JO et al Pediatric cardiovascular care in Uganda: current status, challenges, and opportunities for the future. Ann Pediatr Cardiol 2017;10:50–7. 28163428 10.4103/0974-2069.197069 PMC5241845

[74] Franczyk B , Gluba-Brzozka A, Rysz-Gorzynska M, Rysz J. The role of inflammation and oxidative stress in rheumatic heart disease. Int J Mol Sci 2022;23:15812. DOI: 10.3390/ijms232415812. 36555452 PMC9781220

[75] Passos LSA , Jha PK, Becker-Greene D, Blaser MC, Romero D, Lupieri A et al Prothymosin alpha: a novel contributor to estradiol receptor alpha-mediated CD8(+) T-cell pathogenic responses and recognition of type 1 collagen in rheumatic heart valve disease. Circulation 2022;145:531–48. 35157519 10.1161/CIRCULATIONAHA.121.057301 PMC8869797

[76] Nunes MCP , Barbosa JAA, Mocumbi A. Refining morphological echocardiographic criteria to improve early detection of rheumatic heart disease. Heart 2023;109:1200–1. 37173128 10.1136/heartjnl-2022-322325

[77] Zuhlke L , Karthikeyan G, Engel ME, Rangarajan S, Mackie P, Cupido-Katya Mauff B et al Clinical outcomes in 3343 children and adults with rheumatic heart disease from 14 low- and middle-income countries: two-year follow-up of the Global Rheumatic Heart Disease Registry (the REMEDY Study). Circulation 2016;134:1456–66. 27702773 10.1161/CIRCULATIONAHA.116.024769

[78] Enumah ZO , Boateng P, Bolman RM, Beyersdorf F, Zuhlke L, Musoni M et al Societies of futures past: examining the history and potential of international society collaborations in addressing the burden of rheumatic heart disease in the developing world. Front Cardiovasc Med 2021;8:740745. 34796211 10.3389/fcvm.2021.740745 PMC8592898

[79] Zheleva B , Verstappen A, Overman DM, Ahmad F, Ali SKM, Al Halees ZY et al Advocacy at the eighth world congress of pediatric cardiology and cardiac surgery. Cardiol Young 2023;33:1277–87. 37615116 10.1017/S1047951123002688

